# Thermodynamics in cancers: opposing interactions between PPAR gamma and the canonical WNT/beta-catenin pathway

**DOI:** 10.1186/s40169-017-0144-7

**Published:** 2017-04-12

**Authors:** Yves Lecarpentier, Victor Claes, Alexandre Vallée, Jean-Louis Hébert

**Affiliations:** 1 Centre de Recherche Clinique, Hôpital de Meaux, 6-8 rue Saint Fiacre, 77100 Meaux, France; 2 Department of Pharmaceutical Sciences, University of Antwerp, Wilrijk, Belgium; 3 Experimental and Clinical Neurosciences Laboratory, INSERM U1084, University of Poitiers, Poitiers, France; 4 Institut de Cardiologie, Hôpital de la Pitié-Salpêtrière, Assistance Publique-Hôpitaux de Paris, Paris, France

**Keywords:** PPAR gamma, WNT/beta-catenin, Cancer, Circadian rhythms, Pyruvate dehydrogenase kinase, Pyruvate dehydrogenase complex, Aerobic glycolysis, Warburg effect, PI3 K-AKT pathway, Dissipative structures

## Abstract

Cancer cells are the site of numerous metabolic and thermodynamic abnormalities. We focus this review on the interactions between the canonical WNT/beta-catenin pathway and peroxisome proliferator-activated receptor gamma (PPAR gamma) in cancers and their implications from an energetic and metabolic point of view. In numerous tissues, PPAR gamma activation induces inhibition of beta-catenin pathway, while the activation of the canonical WNT/beta-catenin pathway inactivates PPAR gamma. In most cancers but not all, PPAR gamma is downregulated while the WNT/beta-catenin pathway is upregulated. In cancer cells, upregulation of the WNT/beta-catenin signaling induces dramatic changes in key metabolic enzymes that modify their thermodynamic behavior. This leads to activation of pyruvate dehydrogenase kinase1 (PDK-1) and monocarboxylate lactate transporter. Consequently, phosphorylation of PDK-1 inhibits the pyruvate dehydrogenase complex (PDH). Thus, a large part of pyruvate cannot be converted into acetyl-coenzyme A (acetyl-CoA) in mitochondria and only a part of acetyl-CoA can enter the tricarboxylic acid cycle. This leads to aerobic glycolysis in spite of the availability of oxygen. This phenomenon is referred to as the Warburg effect. Cytoplasmic pyruvate is converted into lactate. The WNT/beta-catenin pathway induces the transcription of genes involved in cell proliferation, i.e., MYC and CYCLIN D1. This ultimately promotes the nucleotide, protein and lipid synthesis necessary for cell growth and multiplication. In cancer, activation of the PI3K-AKT pathway induces an increase of the aerobic glycolysis. Moreover, prostaglandin E2 by activating the canonical WNT pathway plays also a role in cancer. In addition in many cancer cells, PPAR gamma is downregulated. Moreover, PPAR gamma contributes to regulate some key circadian genes. In cancers, abnormalities in the regulation of circadian rhythms (CRs) are observed. CRs are dissipative structures which play a key-role in far-from-equilibrium thermodynamics. In cancers, metabolism, thermodynamics and CRs are intimately interrelated.

## Introduction

Schrödinger in his famous book “What is life” [1] provided us a new understanding of the thermodynamics in living systems. By applying this to the thermodynamics of physical, chemical and biological far-from-equilibrium systems, Prigogine and his colleagues opened new avenues for the exploration of dissipative structures which occupy a major place in the living world [2, 3]. Cancer is an exergonic process in which heat flows from the tumor to its surroundings [4]. The entropy production rate is increased in cancer cells and is characteristic of irreversible processes driven by changes in heat production, Gibbs energy, intracellular acidity, ionic conductance, membrane potential gradient [5]. Numerous cellular mechanisms can induce and develop carcinogenic processes. In most cancers, the WNT/beta-catenin pathway is upregulated while peroxisome proliferator-activated receptor gamma (PPAR gamma) is downregulated. This profile has been observed in several diseases [6] such as cancers [7, 8], type 2 diabetes [9], and certain neurodegenerative diseases (amyotrophic lateral sclerosis [10], Huntington’s disease [11], multiple sclerosis [12, 13] and Friedreich’s ataxia [14]). The opposite profile has been reported in arrhythmogenic right ventricular cardiomyopathy (ARVC) [15, 16], osteoporosis [17–19], and certain neurodegenerative diseases (Alzheimer’s disease [20], Parkinson’s disease [21], bipolar disorder [22, 23] and schizophrenia [24]). From a thermodynamic viewpoint and among numerous cellular processes involved in cancers, two major phenomena play a key role, i.e., aerobic glycolysis or the Warburg effect and disruption of circadian rhythms (CRs). The thermodynamic dysregulation induced by these two processes is consubstantial with metabolic abnormalities commonly found in cancers. PPAR dysfunction influences statistical mechanics by modifying thermodynamic force, thermodynamic flow, and rate of entropy production [5, 25]. We focus our review on the opposing interactions observed in cancers between the canonical WNT/beta-catenin pathway and PPAR gamma and their metabolic and energetic implications.

### Canonical WNT/beta-catenin pathway

**Fig. 1:**
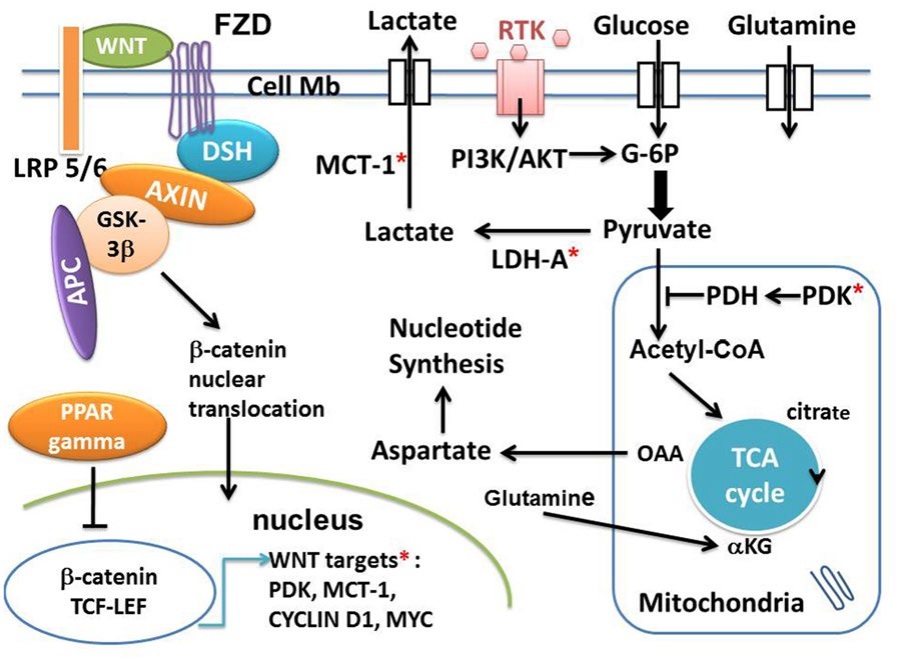
Schema of interactions between the canonical WNT/beta-catenin pathway and PPAR gamma under aerobic glycolysis conditions in cancer. In the absence of the WNT ligands (“off state”), cytosolic beta-catenin is phosphorylated by GSK-3 beta. APS and AXIN combine with GSK-3 beta and beta-catenin to enhance the destruction process in the proteasome. In the presence of the WNT ligands (“on state”), Wnt binds both Frizzled and LRP5/6 receptors to initiate LRP phosphorylation and dishevelled-mediated Frizzled internalization. This leads to dissociation of the AXIN/APC/GSK-3 beta complex. Beta-catenin phosphorylation is inhibited which prevents its degradation in the proteasome. Thus, beta-catenin accumulates in the cytosol and then translocates to the nucleus to bind TCF-LEF co-transcription factors. This induces the WNT-response gene transcription (PDK, MCT-1, MYC, CYCLIN D1). Glucose itself activates the WNT pathway. PPAR gamma inhibits the beta-catenin/TCF-LEF-induced activation of WNT target genes. PDK inhibits the PDH complex in mitochondria. Thus pyruvate cannot be fully converted into acetyl-CoA and enter the TCA cycle. MYC activates LDH-A which converts cytosolic pyruvate into lactate. MCT-1 favors lactate extrusion out of the cytosol which favors angiogenesis. MYC increases glutamine entry in the cytosol and mitochondria. MYC-induced glutamine enhances aspartate and nucleotide synthesis. *APC* adenomatous polyposis coli, *alpha*-*KG* alpha ceto-glutarate, *DSH* Dishevelled, *FZD* Frizzled, *GSK*-*3beta* glycogen synthase kinase-3beta, *LDH* lactate dehydrogenase, *LRP5/6* low-density lipoprotein receptor-related protein 5/6, *MCT*-*1* monocarboxylate lactate transporter-1, OAA: oxalo-acetic acid, *PPAR gamm* peroxisome proliferator-activated receptor gamma, *PDH* pyruvate dehydrogenase complex, *PDK* pyruvate dehydrogenase kinase, *RTK* receptor tyrosine kinase, *TCF/LEF* T-cell factor/lymphoid enhancer factor, *TCA* tricarboxylic acid, *WNT targets: PDK, MCT-1, MYC, CYCLIN D1

**Fig. 2:**
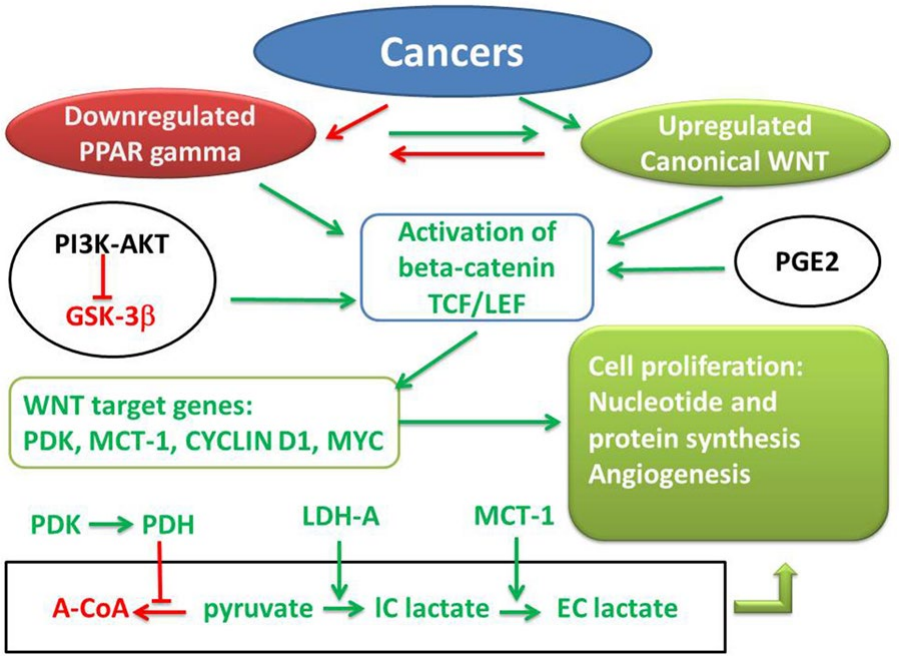
Synthetic diagram of opposing effects of PPAR gamma and canonical WNT/beta-catenin signaling in cancer. *Green arrow* activation; *red arrow* inhibition; *A*-*CoA* acetyl-CoA, *GSK*-*3beta* glycogen synthase kinase-3beta, *IC lactate* intracellular lactate, *EC lactate* extracellular lactate, *GSK*-*3beta* glycogen synthase kinase-3beta, *LDH*-*A* lactico-dehydrogenase-A, *MCT*-*1* monocarboxylate lactate transporter-1, *PI3* *K*-*AKT* phosphatidylinositol 3-kinase-protein kinase B, *PDH* pyruvate dehydrogenase, *PDK* pyruvate dehydrogenase kinase, *TCF/LEF* T-cell factor/lymphoid enhancer factor, *PPAR gamma* peroxisome proliferator-activated receptor gamma

The canonical WNT/beta-catenin pathway plays an important role in metabolism, embryonic development, cell fate, and epithelial-mesenchymal transition (EMT) [ 26 ]. The canonical WNT activity is reflected by elevated levels of beta-catenin in the nucleus and/or cytoplasm, which can be detected by means of immunohistochemical staining, Western blotting and semiquantitative RT-PCR [27]. Its dysfunction is involved in numerous diseases, particularly in cancers [28–31]. The transcription factor beta-catenin/T-cell factor/lymphoid enhancer factor (TCF/LEF) represents the key effector of the canonical WNT pathway (Figs. 1, 2). The destruction complex consists of AXIN, tumor suppressor adenomatous polyposis coli (APC), and glycogen synthase kinase-3 (GSK-3beta). The destruction complex exerts a tight control on the beta-catenin signaling. In the absence of WNT ligands (“off state”), the destruction complex phosphorylates beta-catenin which is then degraded in the proteasome. In the presence of WNT ligands (“on state”), the WNT receptor interacts with Frizzled (FZL) and LDL receptor-related protein 5/6 (LRP5/6). WNT receptor is associated with Dishevelled (DSH). This triggers the disruption of the destruction complex and prevents degradation of beta-catenin in the proteasome. Beta-catenin then translocates to the nucleus and interacts with TCF/LEF. This leads to the stimulation of the beta-catenin target genes (pyruvate dehydrogenase kinase (PDK), monocarboxylate lactate transporter-1 (MTC-1), MYC, CYCLIN D1, cyclooxygenase-2 (COX-2), AXIN) [32–35] (Fig. 1).

### PPAR gamma

Peroxisome proliferator-activated receptor gamma is a ligand-activated transcriptional factor that belongs to the nuclear hormone receptor superfamily [36]. It heterodimerizes with the retinoid X receptor. PPAR gamma is expressed in numerous cell types, such as adipose tissues, muscles, brain, and immune cells. PPAR gamma activates the expression of many genes and regulates glucose homeostasis, insulin sensitivity, lipid metabolism, immune responses, cell fate and inflammation [37–39]. PPAR gamma agonists thiazolidinediones (TZDs) improve insulin sensitivity in peripheral tissues [40] and ameliorate glucose tolerance and insulin sensitivity in type 2 diabetic patients [41]. TZDs act on the promoters of glucose transporter (GLUT-2) and glucokinase (GK) in pancreatic beta-cells and liver. Abnormalities of PPAR gamma are observed in several pathological states such as cancers, diabetes, obesity, and atherosclerosis. Some TZDs have been used for treating type 2 diabetes. PPAR gamma also plays an important role in regulating cardiovascular rhythms by controlling circadian variations of blood pressure and heart rate through BMAL1 [42, 43]. However, numerous side effects induced by TZD have been reported [44].

### Opposing effects of the canonical WNT/beta-catenin pathway and PPAR gamma

The link between the WNT/beta-catenin pathway and PPAR gamma involves the TCF/LEF beta-catenin-binding domain and a catenin binding domain within PPAR gamma. In numerous mammalian cells, PPAR gamma and WNT/beta-catenin signaling behave in an opposite manner [45–50]. In some diseases, although the WNT/beta-catenin pathway is downregulated, PPAR gamma appears to be upregulated and vice versa (see: “Introduction”) [6]. In several cellular systems, beta-catenin is inhibited by PPAR gamma agonists [45, 47, 48, 51]. It has also been observed that inhibition of the WNT/beta-catenin pathway induces activation of PPAR gamma [15].

### Aerobic glycolysis in cancer cells: role of the canonical WNT signaling

The role of the WNT/beta-catenin signaling in cancer development, especially in colorectal cancer, is now better understood [52, 53]. Upregulation of the WNT/beta-catenin pathway via TCF/LEF leads to cell proliferation, EMT, migration and angiogenesis [54–56]. In cancer cells, overactivation of the WNT/beta-catenin pathway induces aerobic glycolysis. This allows glucose utilization for cell proliferation [35]. Thus in a large part, glucose supply is fermented in lactate regardless of oxygen availability. This phenomenon is referred to as aerobic glycolysis or the Warburg effect [57].

In cancer, the behavior of two key enzymes involved in glucose metabolism is modified leading to the Warburg effect. Activation of PDK-1 is required for the Warburg aerobic glycolysis. Upregulation of WNT/beta-catenin signaling activates both PDK-1 and MCT-1 [35, 58]. PDK-1, a major regulator of glucose metabolism, phosphorylates the pyruvate dehydrogenase complex (PDH) which is inhibited and largely prevents the conversion of pyruvate into acetyl-CoA in mitochondria [59]. In colon cancer, PDK-1 is upregulated [35, 60], so that the conversion of pyruvate into acetyl-CoA in mitochondria is diminished with a consequent reduction of acetyl-CoA entering the tricarboxylic acid (TCA) cycle. This induces aerobic glycolysis in spite of the availability of oxygen. PDK-1 has also been observed to be upregulated in several other cancers [61, 62]. Cytosolic pyruvate is converted into lactate through activation of lactic dehydrogenase-A (LDH-A). Upregulation of both LDH-A and MCT-1 results in pyruvate being diverted towards the formation of lactate and the secretion of the latter outside of the cell, which favors angiogenesis [63] and ultimately leads to anabolic production of biomass i.e., nucleotide synthesis [64, 65]. The Warburg effect partly shunts the TCA cycle leading to aerobic glycolysis which is less efficient in terms of ATP production. The most cost effective way producing ATP is via glucose oxidation (ATP/O_2_ = 6.4), since the pathway via free fatty acid beta-oxidation is less efficient (ATP/O_2_ = 5.6). This takes about 11% more O_2_ to produce the same amount of ATP from fatty acids as it does from glucose. Moreover, PDK-1 and 2 enhance angiogenesis [66, 67]. Blocking WNT reduces the PDK-1 level via the transcription regulation and reduces in vivo tumor growth [35]. Conversely, PPAR gamma activation selectively decreases PDK mRNA [68]. PDKs allow metabolic flexibility [69] and are transcriptionally regulated by insulin, glucocorticoids, thyroid hormone and fatty acids [70]. Several diseases presenting PDK abnormalities are often associated with type 2 diabetes, obesity, metabolic disorders, cardiomyopathies, neuropathies and cancers.

In colon cancer, activation of WNT/beta-catenin signaling decreases the oxidative metabolism in the TCA cycle and promotes cell proliferation [35]. In addition, the WNT/beta-catenin pathway induces the transcription of genes involved in cell proliferation, particularly CYCLIN D1 and MYC operating through the G1 phase [71–74]. MYC activates aerobic glycolysis and glutaminolysis and favors nucleotide synthesis [75, 76]. MYC also activates LDH-A, induces glutamine uptake into the cell and mitochondria, and stimulates aspartate synthesis which favors nucleotide synthesis [75] (Fig. 1). Moreover, MYC increases the hypoxia-inducible factor -1alpha (HIF1A) which controls PDK-1 [77]. Part of the pyruvate is converted into acetyl-CoA which in turn enters the TCA cycle and is converted into citrate. This promotes protein and lipid synthesis. Cellular accumulation of metabolic intermediates (aspartate, serine, glycine, and ribose) allows de novo nucleotide synthesis, which contributes to growth and proliferation.

Phosphofructokinase (PFK), an allosteric enzyme, is responsible for glycolytic oscillations. PFK can lead to instabilities beyond which a new state can be organized in time and in space [78]. A positive feedback is responsible for periodic behavior. These far-from-equilibrium oscillatory mechanisms come within the field of dissipative structures initially described by Illia Prigogine [79]. Elevated PFK-1 activity is characteristic of cancer cells and is induced in response to oncogenes [80].

Cancer cells are characterized by increased glucose consumption. High serum glucose levels may modulate cancer-related processes. Glucose itself can directly impact the canonical WNT pathway [81]. High glucose level enhances the nuclear translocation of beta-catenin in response to WNT activation. In cancer cells, glucose-induced beta-catenin acetylation favors the WNT pathway.

### Aerobic glycolysis and vitamin C

It has been recently described a novel antitumoral mechanism of vitamin C [82]. Mutation of the proto-oncogene KRAS is often present in colon and pancreatic cancer. In KRAS mutant colorectal cancer, this mechanism involves the Warburg metabolic disruption. In the absence of vitamin C, pyruvate kinase PKM2 is phosphorylated, then translocates to the nucleus and binds the beta-catenin/TCF/LEF transcriptional factor. This promotes the MYC transcription which in turn enhances GLUT-1 and Polypyrimidine Tract Binding Protein (PTB) expression. In the presence of vitamin C which enters into the cell via GLUT-1, RAS is detached from the cell membrane which blocks the PKM2 phosphorylation. This induces downregulation of GLUT-1 and PKM2 expression via disruption of the beta-catenin/TEF/LEF transcriptional complex. This leads to downregulation of MYC and inhibition of the Warburg pathway. Thus, vitamin C uncouples the Warburg metabolic switch in KRAS mutant colon cancer.

### Thermodynamics and lawless-disorderly cancer growth

From a thermodynamic viewpoint, the lawless-disorderly cancer growth and the orderly fetal growth share some similar features [83]. Hypoxic conditions reported in cancer cells for their growth requirements resemble to those observed during normal fetal growth, which requires a relatively low oxygen tension. For both cancerous and fetal growth, low energy requirements are linked to the tumorigenic arm of acute inflammation [83], as in wound healing. Moreover, the production of lactate under aerobic glycolysis conditions is characteristic of the human placenta [84], a tissue in which the population of contractile myofibroblasts is important [85]. In cancer (mammary carcinoma, epithelial cells in cancerous mammary glands), fibrotic lesions (Dupuytrens nodules, hypertrophic scars) [86], and normal placental stem villi [87], the main myosin molecular motor in myofibroblasts is the non muscle myosin (NMM). Kinetics of contractile NMM crossbridges are dramatically slow [88] and their entropy production rate is extremely low [89]. The presence of numerous myofibroblasts is associated with the aerobic glycolysis metabolism. In epithelial cancers, myofibroblasts represent a significant part of the stroma reaction. Myofibroblasts, epithelial cells, and connective tissue cells participate to cancer invasion, with loss of epithelial characteristics and acquisition of mesenchymal properties. This refers to as EMT [26] which greatly influences the invasive carcinoma progression and in which the canonical WNT pathway plays a key role. WNT3a favors myofibroblast differentiation by upregulating the transforming growth factor (TGF-beta1). This occurs through SMAD2 in a beta-catenin-dependent manner [90]. Importantly, it has been recently demonstrated that aerobic glycolysis is induced in response to TGF-beta1 [91].

### Activation of WNT/beta-catenin pathway and inactivation of PPAR gamma in cancers

WNT/beta-catenin signaling has been found to be activated in cancers [92, 93]. WNT1 was first discovered as a proto-oncogene in a breast cancer mouse model. Increased expression of beta-catenin may be due to factors such as mutations in beta-catenin, abnormalities in the beta-catenin destruction complex, mutations in APC, overexpression of WNT ligands, and loss of inhibition or decreased activity of regulatory pathways. Alterations in gene expression of *CTNNB1* which encodes beta-catenin, have been reported in numerous cancers such as breast colorectal, melanoma, prostate and lung tumors. WNT 1, WNT2 and WNT7A ligand-proteins are overexpressed in glioblastoma, esophageal cancer and ovarian cancer respectively. Proteins of the TCF/LEF family and WNT5A may also induce cancer. Repression of WNT/beta-catenin signaling can prevent EMT and inhibit metastasis. Mutations of the WNT pathway components are associated with many cancers, particularly with colorectal cancer. APC deficiency and beta-catenin mutations upregulate the WNT/beta-catenin pathway and prevent beta-catenin degradation. This leads to excessive stem cell renewal and cell proliferation that predisposes to tumor genesis particularly for colorectal cancer [94]. Nuclear accumulation of beta-catenin drives cancer cell proliferation. In colon cancer, beta-catenin-TCF/LEF signaling is activated [95], and activation of the WNT pathway via *APC* gene mutations favors cell proliferation [96]. Mutations in PPAR gamma are linked with human colon cancer [97].

Several studies have presented evidence for a protective role of PPAR gamma against cancer. In colon cancer, PPAR gamma downregulates the oncogene beta-catenin and suppresses cell proliferation [98]. In contrast, other studies have implicated PPAR gamma in the promotion and development of cancer [8]. Thus, PPAR gamma activation by specific agonists can induce growth inhibition, apoptosis and differentiation of numerous tumor cells. On the contrary, overexpression of PPAR gamma has been reported in tumors of colon, breast, prostate, stomach, salivary gland, cervix, ovary, bladder, lung, testes and the neural crest element of sympathetic nervous system [7]. The biological significance of PPAR gamma in cancer remains controversial. Activation of PPAR gamma can induce either tumor suppressive or promoting responses. On the one hand, PPAR gamma can act as a tumor inhibitor in colon cancer [99–105], in breast cancer [106–110], in urological cancer [110–115], in lung cancer [116–118], and in gastric cancer [119–122]. On the other hand, PPAR gamma can act as a tumor promotor in colon cancer [123–126], in breast cancer [127–132], and in urological cancer [133–135]. There is no clear unifying accepted mechanism explaining these contradictory evidences concerning either the protective role of PPAR gamma or their role on promotion/development of cancer. This might be partly explained by cell type-specific effects, organ-specific effects, receptor-independent effects according to the PPAR gamma agonist used. This might also be due to specific pharmacokinetic properties of PPAR gamma ligands or the stage of cancer development at which the PPAR gamma ligand is administered [8]. These arguments are hypotheses, and for the time being, no universal mechanism is able to explain the contradictory effects of PPAR gamma ligands on cancers.

### Role of PI3K-AKT pathway in aerobic glycolysis and cancers

Hyperactivation of phosphatidylinositol 3-kinase (PI3K)-protein kinase B (AKT) pathway is associated with an increased rate of glucose metabolism in tumor cells [136]. AKT signaling directly acts on aerobic glycolysis in cancer cells. AKT regulates the localization of GLUT1 in the plasma membrane and hexokinase expression. It also activates phosphofructokinase-1 (PFK-1) which directly phosphorylates PFK-2. This leads to produce fructose-2.6-bisphosphate, an activator of PFK-1. AKT activation causes an increase in aerobic glycolysis or Warburg effect in cancer. PI3K-AKT pathway promotes cell survival, cell growth, cell proliferation, cell migration and angiogenesis in response to extracellular signals including hormones and growth factors. This pathway is stimulated by the binding of extracellular ligands to a receptor tyrosine kinase (RTK) located in the plasma membrane (Fig. 1). This signaling is upregulated in certain cancers. Through phosphorylation of GSK-3beta, PI3 K-AKT favors the G1 phase of the cell cycle. GSK-3beta phosphorylation decreases the degradation of beta-catenin in the proteasome. Thus, TCF/LEF transcription factor is activated which in turn favors transcription of the target gene CYCLIN D1 [137]. Consequently, by decreasing the GSK-3beta activity, AKT pathway behaves similarly to the WNT pathway. Aberrant activation of PI3K-AKT is often associated with cancers, including glioblastomas, ovarian, pancreatic and breast cancers [138]. AKT mRNA is increased in breast and prostate cancer. PI3K-AKT contributes to angiogenesis by acting on the vascular endothelial growth factor in endothelial cells and on the endothelial nitric oxide synthase. This activates vasodilation and vascular remodeling [139]. Moreover, the PI3K-AKT pathway increases the hypoxia-inducible transcription factor [140].

The phosphatase and tensin homologue (PTEN) represents the main brake of the PI 3′-OH kinase (PIK3)-AKT pathway [141]. PI3K generate phosphatidylinositol-3,4,5-triphosphate (PIP3) from PIP2. AKT is activated by PIP3. PTEN is a PIP3-phosphatase and its activity is opposed to that of PI3K. PI3K-AKT signaling is a major pathway which is activated in cancer. PTEN appears to be relevant against cancer progression and represents a target for somatic cancer inactivation. In some cancers (endometrial, breast, and colorectal cancers), PI3K and PTEN mutations coexist. PTEM also induces a decrease in cancer cell proliferation due to cell cycle arrest in the G1 phase.

### Prostaglandins, WNT and PPAR gamma

Several studies have established the role of prostaglandin E2 (PGE2) by activating the WNT/beta-catenin pathway. The link between PGE2 and the canonical WNT pathway suggests that chronic inflammation induced by a prolonged increase of PGE2 could lead to activation of WNT signaling resulting in cell proliferation and cancer. PGE2 enhances the beta-catenin-dependent transcription [142, 143]. PGE2 promotes colon cancer cell growth through the beta-catenin pathway. Thus, blockage of WNT/beta-catenin signaling can be of interest for cancer treatment. In treatment of colorectal cancer, nonsteroidal anti-inflammatory drugs (NSAIDs) induce beneficial effects [144], partly due to their interaction with the beta-catenin pathway and their inhibition of the PGE2 synthesis. PGE2 modulates the WNT activity in hematopoietic stem cell (HSC) in zebrafish. Inhibition of PGE2 synthesis blocks alterations in HSC induced by WNT. PGE2 modifies the WNT signaling cascade at the level of beta-catenin degradation through the cAMP/PKA pathway. WNT activation in stem cells requires PGE2 [145]. Dimethyl-prostaglandin E2 increases HSC in vivo. In addition, dimethyl-prostaglandin E2 leads to the formation of components of the WNT pathway [146]. WNT signaling upregulates interleukin (IL)-7R and IL-2Rbeta. In neuroectodermal (NE-4C) stem cells, PGE2 interacts with the canonical WNT signaling through PKA and PI3K [147]. In WNT-induced cells, beta-catenin is increased and the WNT-target genes (*Ctnnb1*, *Ptgs2*, *Ccnd1*, *Mmp9*) are significantly upregulated after PGE2 use. PPAR gamma and proinflammatory enzyme pathways are interrelated. Decreased expression of PPAR gamma and high levels of COX-2 have been reported in many cancers [148]. TZDs decrease COX-2, inhibit growth of non-small-cell lung cancer cells in vitro, and block tumor development. TZDs diminish COX-2 and PGE2 through PPAR gamma. The PPAR gamma activator 15dPGJ2 plays an anti-inflammatory role in a PPAR gamma-dependent manner, decreasing COX-2, PGE2 and iNos expression [149].

### Circadian rhythms (CRs), cancers, metabolism and thermodynamics

CRs can be defined as endogenous, entrainable free-running periods that last approximately 24 h. CRs are far-from-equilibrium dissipative structures and are due to a negative feedback produced by a protein on the expression of its own gene [150–152]. They operate in far-from-equilibrium manner if affinity of the studied system is ≫RT (R is the universal gas constant and T is the absolute temperature), and generate order spontaneously by exchanging energy with their external environment [2, 153]. In mammals, CRs involve several major critical transcription factors such as circadian locomotor output cycles kaput (CLOCK), brain and muscle aryl-hydrocarbon receptor nuclear translocator-like1 (BMAL1), period 1 (PER1), period 2 (PER2), and period 3 (PER3) [154, 155]. Transcription/translation autoregulatory feedback loops with both activating and inhibiting pathways are involved in CRs [156, 157].

Circadian rhythms govern numerous physiological and metabolic functions [158]. Thus, CRs are observed in sleep-awake and feeding patterns, energy metabolism, body temperature, hormone secretion, heart rate and blood pressure. Following epidemioloigical and genetic probes, it has been suggested that disruption of CRs may be directly linked to cancer, leading to aberrant cellular proliferation [159]. Since numerous connections between the circadian clock and cellular metabolism have been reported, it is thougth that the abnormal metabolism observed in cancer may be a consequence of disrupted CRs. CRs within the cell regulate the timing of many important life cycles [160]. The phase diffusion constant depends on the free-energy dissipation per period. Oscillations are driven by multiple irreversible cycles that hydrolyze fuel molecules such as ATP. The free energy consumed per period is proportional to the number of phase coherent periods. A decreased *BMAL1* function modifies the behavior of genes involved in the canonical WNT pathway [161]. Beta-catenin induces PER2 degradation altering circadian clock gene in intestinal mucosa of ApcMin/+ mice [162]. A deceased expression level of PER1 and/or PER2 has been reported in numerous cancers: breast cancer [163], prostate cancer [164], pancreatic cancer [165], colorectal cancer [166], chronic myeloid leukemia [167], and glioma [168, 169].

Peroxisome proliferator-activated receptors interferes with the mammalian clock and energy metabolism [170]. PPARs are rhythmically expressed in mammalian tissues [171] and directly interact with the core clock genes. PPAR gamma exhibits variations in diurnal expression in mouse fat, liver and blood vessels [42]. Deletion of *PPAR gamma* in mouse impairs diurnal rhythms [172]. PPAR gamma plays an important role in the coordinated control of circadian clocks, metabolism and cardiac performance. PGC-1 alpha, a transcriptional co-activator that regulates energy metabolism, is rhythmically expressed in the liver and skeletal muscle of mice. PGC-1 alpha upregulates the expression of the clock genes *BMAL1* and *Rev*-*erb alpha*. Mice lacking PGC-1 alpha show changes in CRs and metabolism [173]. PGC-1 alpha acts as a stress sensor in cancer cells. In maintaining metabolic homeostasis, PGC-1 alpha favors cancer cell survival [174]. PGC-1 alpha interfers in a very complex manner with nuclear receptors such as Rev-erb, ROR, PPARs [175]. PPAR alpha and gamma up-regulate the expression of Rev-erb alpha and BMAL1 by binding to their promotors. PGC-1 potentiates ROR alpha transcriptional activity and enhances both Rev-erb alpha and BMAL1 transcription. Moreover after serum shock, GSK-3beta-mediated stabilization of Rev-erb alpha plays a key role to initiate, maintain and synchronize CRs.

## Conclusions

Cancers exhibit thermodynamic and metabolic alterations and abnormal CRs. In many cancers but not all, the canonical WNT/beta-catenin pathway is upregulated, while PPAR gamma is downregulated, the two systems behaving in an opposite manner. Overactivation of the WNT pathway results in cell proliferation due to the activation of certain target genes of beta-catenin, such as MYC and CYCLIN D1. This promotes protein synthesis and angiogenesis. *PDK* and *MCT*-*1* are also target genes of beta-catenin, explaining the significant decrease in the transformation of pyruvate into acetyl-CoA in mitochondria and the formation of intracellular lactate, which will be extruded out of the cell. This is referred to as aerobic glycolysis or the Warburg phenomenon. The expression of PPAR gamma is decreased due to the overactivation of WNT/beta-catenin signaling. Circadian rhythms, dissipative structures which are governed by the laws of far-from-equilibrium thermodynanics are disrupted in cancers. They are influenced by both the WNT/beta-catenin pathway and PPAR gamma. Changes in thermodynamics, metabolism and circadian rhythms are tightly linked in cancers.

## Abbreviations

acetyl-CoA: acetyl-coenzyme A
APC: adenomatous polyposis coli
ARVC: arrhythmogenic right ventricular dysplasia/cardiomyopathy
BMAL1: brain and muscle aryl-hydrocarbon receptor nuclear translocator-like1
CLOCK: circadian locomotor output cycles kaput
COX-2: cyclooxygenase-2
DSH: dishevelled
EMT: epithelial-mesenchymal transition
FZD: frizzled
GK: glucokinase
GLUT: glucose transporter
GSK-3beta: glycogen synthase kinase-3beta
HSC: hematopoietic stem cell
LDH: lactate dehydrogenase
LRP5/6: low-density lipoprotein receptor-related protein 5/6
MCT-1: monocarboxylate lactate transporter-1
NSAID: nonsteroidal anti-inflammatory drug
PER: period
PPAR: peroxisome proliferator-activated receptor
PGC-1 alpha: peroxisome proliferator-activated receptor-gamma coactivator-1 alpha
PI3K-AKT: phosphatidylinositol 3-kinase-protein kinase B
PFK-1: phosphofructokinase-1
PGE2: prostaglandin E2
PDH: pyruvate dehydrogenase complex
PDK: pyruvate dehydrogenase kinase
RTK: receptor tyrosine kinase
TCF/LEF: T cell factor factor/lymphoid enhancer factor
TZD: thiazolidinedione
TGF-beta1: transforming growth factor
TCA: tricarboxylic acid
